# 2,2′-[(4,6-Dinitro-1,3-phenyl­ene)dioxy]diacetic acid hemihydrate

**DOI:** 10.1107/S1600536809036253

**Published:** 2009-09-12

**Authors:** Dong-Sheng Ma, Xiu-Mei Zhang, Hua Zhang, Dan Mu, Guang-Feng Hou

**Affiliations:** a College of Chemistry and Materials Science, Heilongjiang University, Harbin 150080, People’s Republic of China

## Abstract

The skeletons of both independent mol­ecules of the carboxylic acid hemihydrate, C_10_H_8_N_2_O_10_·0.5H_2_O, are approximately planar [maximum deviations 0.642 (3) and 0.468 (1) Å]. The deviations arise from the twisting of the nitro groups with respect to the aromatic rings [dihedral angles = 3.24 (2) and 27.01 (1), and 7.87 (1) and 16.37 (2)° in the two molecules]. The crystal structure features inter­molecular O—H⋯O hydrogen bonds, which the link the dicarboxylic acid and water mol­ecules into a supra­molecular layer network.

## Related literature

For general background to the use of flexible aromatic carboxylic acid ligands, see: Coronado *et al.* (2000 ▶). For the synthesis and related structures, see: Gao *et al.* (2006 ▶).
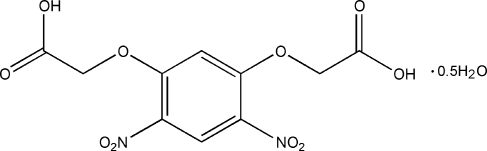


## Experimental

### 

#### Crystal data

C_10_H_8_N_2_O_10_·0.5H_2_O
*M* *_r_* = 325.19
Monoclinic, 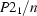

*a* = 7.3873 (15) Å
*b* = 25.918 (5) Å
*c* = 13.711 (3) Å
β = 99.43 (3)°
*V* = 2589.7 (9) Å^3^
*Z* = 8
Mo *K*α radiation
μ = 0.16 mm^−1^
*T* = 291 K
0.25 × 0.21 × 0.20 mm

#### Data collection

Rigaku RAXIS-RAPID diffractometer
Absorption correction: multi-scan (*ABSCOR*; Higashi, 1995 ▶) *T*_min_ = 0.962, *T*_max_ = 0.970
19619 measured reflections
4552 independent reflections
3197 reflections with *I* > 2σ(*I*)
*R*_int_ = 0.046

#### Refinement

*R*[*F* ^2^ > 2σ(*F* ^2^)] = 0.054
*wR*(*F* ^2^) = 0.172
*S* = 1.03
4552 reflections
410 parameters
H-atom parameters constrained
Δρ_max_ = 0.42 e Å^−3^
Δρ_min_ = −0.30 e Å^−3^

### 

Data collection: *RAPID-AUTO* (Rigaku 1998 ▶); cell refinement: *RAPID-AUTO*; data reduction: *CrystalClear* (Rigaku/MSC, 2002 ▶); program(s) used to solve structure: *SHELXS97* (Sheldrick, 2008 ▶); program(s) used to refine structure: *SHELXL97* (Sheldrick, 2008 ▶); molecular graphics: *SHELXTL* (Sheldrick, 2008 ▶); software used to prepare material for publication: *SHELXL97*.

## Supplementary Material

Crystal structure: contains datablocks I. DOI: 10.1107/S1600536809036253/ng2635sup1.cif

Structure factors: contains datablocks I. DOI: 10.1107/S1600536809036253/ng2635Isup2.hkl

Additional supplementary materials:  crystallographic information; 3D view; checkCIF report

## Figures and Tables

**Table 1:** Hydrogen-bond geometry (Å, °)

*D*—H⋯*A*	*D*—H	H⋯*A*	*D*⋯*A*	*D*—H⋯*A*
O2—H2⋯O11^i^	0.82	1.89	2.698 (3)	168
O4—H5⋯O21^ii^	0.82	1.74	2.562 (3)	174
O12—H12⋯O1^iii^	0.82	1.92	2.730 (3)	168
O14—H15⋯O18^iv^	0.82	2.20	2.921 (3)	147
O14—H15⋯O17^iv^	0.82	2.37	3.055 (3)	141
O21—H21⋯O15	0.85	2.19	2.947 (4)	148
O21—H21⋯O10	0.85	2.66	3.084 (4)	113
O21—H22⋯O5	0.85	1.97	2.798 (4)	165

Symmetry codes: (i) 
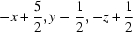
; (ii) 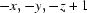; (iii) 
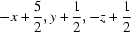
; (iv) 
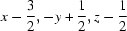
.

**Fig. 1.**
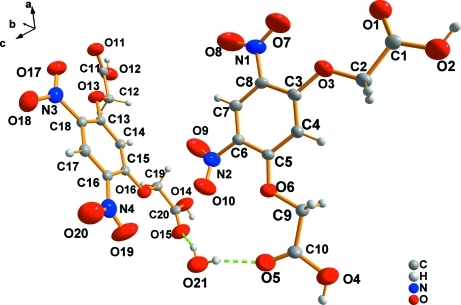
The molecular structure of the title compound, showing displacement ellipsoids at the 50% probability level for non-H atoms.

**Fig. 2.**
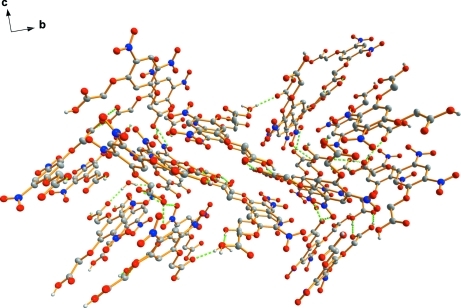
A partial packing view, showing the three-dimensional supramolecular network. Dashed lines indicate the hydrogen-bonding interactions and no involving H atoms have been omitted.

## supplementary crystallographic information

### Comment

Flexible aromatic carboxylic acid with oxygen is a kind of biological activity of the organic carboxylic acid, not only in agriculture, such as plant growth regulators and herbicides it is applied, but also it is important to the synthesis of some organic medicine centre body. Compared with other rigid carboxylic acid ligands, such flexible aromatic carboxylic acid have highly plasticity and spatial configuration of, so it provides a rich and colorful way to identify and assemble for constructing a novel topological network structure with the special physical and chemical properties (Coronado *et al.*, 2000; Gao *et al.*, 2006). In this paper, we report the synthesis and crystal structures of a new flexible aromatic carboxylic acid compound.

In the crystal structure, the skeletons of the two dicarboxylic acid molecules are all approximately co-planar with the largest deviation being 0.642 (3) Å, 0.4681 Å from O8 and O15 for molecule C1–C10 and molecule C11–C20, respectively (Figure 1). This deviations are caused by the twisting of the nitro groups with the benzene planes.

There are six symmetry-independent 'active' H atoms in the crystal structure, all of them participate in hydrogen bonds, which link the dicarboxylic acid and water molecules into a two-dimensional layer supramolecular network (Table 1, Figure 2).

### Experimental

The synthesis of target product is as follows: chlorine acetic acid (51.6 g, 0.54 mol), sodalye(21.8 g, 0.54 mol) were dissolved into 200 ml distilled water with stirring. The mixture was heated to refluxed for 6 h, then the pH value was adjusted to about 2.0 by using 3 *M* hydrochloric acid. After cooling to the room temperature, 10.8 g (27%) yellow precipitate was obtained. The 10.8 g above yellow product was dissolved into 100 ml concentrated sulfuric acid with stirring, and then the mixture of nitric acid (9.45 g, 0.15 mol) and sulfuric acid (20.58 g, 0.21 mol) was dropped into the above solution with keeping the reaction trmperature under 0 ° C for 1 h. The mixture was poured into 500 ml water solution. The crude product was recrystallized from 100 ml water solution, 4.2 g yellow needle crystal was obtained (30%).

### Refinement

H atoms bound to C atoms were placed in calculated positions and treated as riding on their parent atoms, with C—H = 0.93 Å (aromatic), C—H = 0.97 Å (methylene), and with *U*_iso_(H) = 1.2*U*_eq_(C). Water H atoms were initially located in a difference Fourier map, but they were treated as riding on their parent atoms with O—H = 0.85 Å and with with *U*_iso_(H) = 1.5 *U*_eq_(O). Carboxylic H atoms were found in a difference Fourier map, and refined with *U*_iso_(H) = 1.5 *U*_eq_(O).

### Crystal data

**Table:**

C_10_H_8_N_2_O_10_·0.5H_2_O	*F*(000) = 1336
*M**_r_* = 325.19	*D*_x_ = 1.668 Mg m^−^^3^
Monoclinic, *P*2_1_/*n*	Mo *K*α radiation, λ = 0.71073 Å
Hall symbol: -P 2yn	Cell parameters from 13902 reflections
*a* = 7.3873 (15) Å	θ = 3.0–27.4°
*b* = 25.918 (5) Å	µ = 0.16 mm^−^^1^
*c* = 13.711 (3) Å	*T* = 291 K
β = 99.43 (3)°	Block, colorless
*V* = 2589.7 (9) Å^3^	0.25 × 0.21 × 0.20 mm
*Z* = 8	

### Data collection

**Table:**

Rigaku RAXIS-RAPID diffractometer	4552 independent reflections
Radiation source: fine-focus sealed tube	3197 reflections with *I* > 2σ(*I*)
graphite	*R*_int_ = 0.046
ω scan	θ_max_ = 25.0°, θ_min_ = 3.0°
Absorption correction: multi-scan (*ABSCOR*; Higashi, 1995)	*h* = −8→8
*T*_min_ = 0.962, *T*_max_ = 0.970	*k* = −30→30
19619 measured reflections	*l* = −16→16

### Refinement

**Table:**

Refinement on *F*^2^	Primary atom site location: structure-invariant direct methods
Least-squares matrix: full	Secondary atom site location: difference Fourier map
*R*[*F*^2^ > 2σ(*F*^2^)] = 0.054	Hydrogen site location: inferred from neighbouring sites
*wR*(*F*^2^) = 0.172	H-atom parameters constrained
*S* = 1.03	*w* = 1/[σ^2^(*F*_o_^2^) + (0.099*P*)^2^ + 0.8965*P*] where *P* = (*F*_o_^2^ + 2*F*_c_^2^)/3
4552 reflections	(Δ/σ)_max_ < 0.001
410 parameters	Δρ_max_ = 0.42 e Å^−^^3^
0 restraints	Δρ_min_ = −0.29 e Å^−^^3^

### Special details

**Table:**

Geometry. All e.s.d.'s (except the e.s.d. in the dihedral angle between two l.s. planes) are estimated using the full covariance matrix. The cell e.s.d.'s are taken into account individually in the estimation of e.s.d.'s in distances, angles and torsion angles; correlations between e.s.d.'s in cell parameters are only used when they are defined by crystal symmetry. An approximate (isotropic) treatment of cell e.s.d.'s is used for estimating e.s.d.'s involving l.s. planes.
Refinement. Refinement of *F*^2^ against ALL reflections. The weighted *R*-factor *wR* and goodness of fit *S* are based on *F*^2^, conventional *R*-factors *R* are based on *F*, with *F* set to zero for negative *F*^2^. The threshold expression of *F*^2^ > σ(*F*^2^) is used only for calculating *R*-factors(gt) *etc*. and is not relevant to the choice of reflections for refinement. *R*-factors based on *F*^2^ are statistically about twice as large as those based on *F*, and *R*- factors based on ALL data will be even larger.

### Fractional atomic coordinates and isotropic or equivalent isotropic displacement parameters (Å^2^)

**Table:**

	*x*	*y*	*z*	*U*_iso_*/*U*_eq_	
C1	1.0310 (4)	−0.05466 (12)	0.0762 (2)	0.0453 (7)	
C2	0.8978 (5)	−0.03824 (14)	0.1436 (3)	0.0567 (9)	
H2A	0.7756	−0.0337	0.1062	0.068*	
H2B	0.8927	−0.0639	0.1946	0.068*	
C3	0.8704 (4)	0.03522 (12)	0.2451 (2)	0.0424 (7)	
C4	0.7138 (4)	0.01642 (12)	0.2754 (2)	0.0455 (8)	
H4	0.6713	−0.0162	0.2548	0.055*	
C5	0.6181 (4)	0.04510 (11)	0.3361 (2)	0.0393 (7)	
C6	0.6824 (4)	0.09461 (11)	0.3636 (2)	0.0416 (7)	
C7	0.8398 (4)	0.11370 (11)	0.3348 (2)	0.0442 (7)	
H7	0.8811	0.1466	0.3544	0.053*	
C8	0.9353 (4)	0.08438 (11)	0.2776 (2)	0.0415 (7)	
C9	0.3957 (4)	−0.02105 (11)	0.3406 (3)	0.0486 (8)	
H9A	0.4864	−0.0476	0.3611	0.058*	
H9B	0.3611	−0.0226	0.2693	0.058*	
C10	0.2310 (4)	−0.02900 (12)	0.3895 (3)	0.0481 (8)	
C11	1.0980 (4)	0.38191 (11)	0.5469 (2)	0.0425 (7)	
C12	0.9397 (4)	0.34925 (11)	0.5686 (2)	0.0423 (7)	
H12A	0.8560	0.3699	0.6000	0.051*	
H12B	0.8726	0.3349	0.5080	0.051*	
C13	0.9082 (4)	0.27089 (11)	0.6568 (2)	0.0358 (6)	
C14	0.7269 (4)	0.26400 (11)	0.6126 (2)	0.0368 (6)	
H14	0.6759	0.2870	0.5636	0.044*	
C15	0.6187 (3)	0.22418 (10)	0.6385 (2)	0.0327 (6)	
C16	0.6953 (4)	0.19013 (10)	0.7146 (2)	0.0354 (6)	
C17	0.8751 (4)	0.19648 (11)	0.7588 (2)	0.0372 (7)	
H17	0.9256	0.1737	0.8083	0.045*	
C18	0.9820 (4)	0.23571 (11)	0.7313 (2)	0.0370 (7)	
C19	0.3662 (4)	0.25045 (11)	0.5203 (2)	0.0385 (7)	
H19A	0.4391	0.2513	0.4676	0.046*	
H19B	0.3623	0.2850	0.5472	0.046*	
C20	0.1761 (4)	0.23215 (12)	0.4812 (2)	0.0433 (7)	
N1	1.1037 (4)	0.10698 (12)	0.2527 (2)	0.0560 (7)	
N2	0.5870 (4)	0.12895 (10)	0.4221 (2)	0.0542 (7)	
N3	1.1684 (3)	0.23969 (10)	0.78400 (19)	0.0430 (6)	
N4	0.5927 (4)	0.14953 (9)	0.75239 (19)	0.0444 (6)	
O1	1.1678 (3)	−0.03181 (8)	0.06594 (18)	0.0536 (6)	
O2	0.9765 (3)	−0.09865 (9)	0.03349 (19)	0.0604 (7)	
H2	1.0469	−0.1072	−0.0041	0.091*	
O3	0.9670 (3)	0.00887 (9)	0.18550 (19)	0.0589 (7)	
O4	0.1546 (4)	−0.07382 (9)	0.3654 (2)	0.0720 (8)	
H5	0.0629	−0.0771	0.3914	0.108*	
O5	0.1750 (3)	0.00126 (10)	0.4427 (2)	0.0687 (8)	
O6	0.4686 (3)	0.02851 (8)	0.36993 (17)	0.0509 (6)	
O7	1.1969 (5)	0.08272 (13)	0.2060 (3)	0.1029 (12)	
O8	1.1449 (5)	0.14971 (13)	0.2799 (4)	0.1298 (17)	
O9	0.6063 (5)	0.17479 (10)	0.4134 (3)	0.0998 (12)	
O10	0.4939 (4)	0.11086 (10)	0.4787 (3)	0.0919 (11)	
O11	1.2585 (3)	0.36932 (9)	0.56650 (17)	0.0533 (6)	
O12	1.0395 (3)	0.42478 (9)	0.5016 (2)	0.0614 (7)	
H12	1.1271	0.4410	0.4877	0.092*	
O13	1.0174 (3)	0.30879 (8)	0.63347 (16)	0.0463 (5)	
O14	0.0992 (3)	0.26513 (11)	0.4130 (2)	0.0704 (8)	
H15	−0.0088	0.2573	0.3954	0.106*	
O15	0.1017 (3)	0.19503 (9)	0.5074 (2)	0.0635 (7)	
O16	0.4453 (2)	0.21590 (7)	0.59524 (14)	0.0391 (5)	
O17	1.2814 (3)	0.26610 (9)	0.75223 (18)	0.0541 (6)	
O18	1.2051 (3)	0.21598 (11)	0.86301 (18)	0.0644 (7)	
O19	0.4279 (4)	0.14545 (11)	0.7251 (2)	0.0824 (9)	
O20	0.6744 (4)	0.12010 (10)	0.8133 (2)	0.0701 (7)	
O21	0.1450 (4)	0.08578 (9)	0.5660 (2)	0.0706 (8)	
H21	0.1745	0.1146	0.5437	0.106*	
H22	0.1744	0.0616	0.5298	0.106*	

### Atomic displacement parameters (Å^2^)

**Table:**

	*U*^11^	*U*^22^	*U*^33^	*U*^12^	*U*^13^	*U*^23^
C1	0.0422 (17)	0.0467 (17)	0.0464 (19)	0.0058 (14)	0.0056 (14)	−0.0020 (15)
C2	0.050 (2)	0.061 (2)	0.064 (2)	0.0008 (16)	0.0214 (17)	−0.0124 (18)
C3	0.0395 (16)	0.0499 (17)	0.0399 (17)	0.0061 (13)	0.0127 (14)	0.0010 (14)
C4	0.0448 (18)	0.0434 (17)	0.0512 (19)	−0.0052 (13)	0.0161 (15)	−0.0092 (14)
C5	0.0367 (16)	0.0395 (16)	0.0446 (17)	−0.0044 (12)	0.0150 (14)	−0.0017 (13)
C6	0.0417 (17)	0.0380 (16)	0.0481 (18)	−0.0023 (12)	0.0164 (14)	−0.0013 (13)
C7	0.0448 (17)	0.0366 (15)	0.0530 (19)	−0.0034 (13)	0.0137 (15)	0.0028 (14)
C8	0.0369 (16)	0.0439 (16)	0.0450 (18)	−0.0030 (12)	0.0103 (14)	0.0060 (14)
C9	0.0491 (18)	0.0380 (16)	0.063 (2)	−0.0102 (14)	0.0229 (16)	−0.0099 (15)
C10	0.0440 (18)	0.0471 (18)	0.056 (2)	−0.0073 (14)	0.0173 (16)	−0.0031 (16)
C11	0.0497 (19)	0.0412 (16)	0.0380 (17)	0.0002 (14)	0.0117 (14)	−0.0012 (13)
C12	0.0350 (16)	0.0485 (17)	0.0439 (18)	0.0029 (13)	0.0076 (13)	0.0062 (14)
C13	0.0329 (14)	0.0438 (16)	0.0311 (15)	−0.0027 (12)	0.0063 (12)	−0.0004 (12)
C14	0.0311 (14)	0.0457 (16)	0.0323 (15)	0.0034 (12)	0.0018 (12)	0.0060 (12)
C15	0.0294 (14)	0.0392 (15)	0.0288 (14)	0.0033 (11)	0.0026 (11)	−0.0027 (12)
C16	0.0347 (15)	0.0413 (15)	0.0302 (15)	−0.0010 (12)	0.0054 (12)	−0.0036 (12)
C17	0.0383 (16)	0.0424 (16)	0.0296 (15)	0.0054 (13)	0.0017 (12)	0.0039 (12)
C18	0.0302 (14)	0.0486 (17)	0.0309 (15)	0.0055 (12)	0.0010 (12)	−0.0008 (13)
C19	0.0275 (14)	0.0447 (16)	0.0423 (17)	0.0020 (12)	0.0024 (13)	0.0064 (13)
C20	0.0322 (15)	0.0522 (18)	0.0440 (18)	0.0007 (13)	0.0018 (13)	0.0071 (15)
N1	0.0427 (15)	0.0607 (18)	0.069 (2)	−0.0023 (13)	0.0232 (15)	0.0033 (15)
N2	0.0590 (17)	0.0394 (15)	0.071 (2)	−0.0066 (12)	0.0303 (16)	−0.0094 (14)
N3	0.0350 (13)	0.0550 (16)	0.0370 (15)	0.0061 (12)	−0.0002 (11)	−0.0025 (12)
N4	0.0571 (17)	0.0395 (14)	0.0356 (14)	0.0016 (12)	0.0045 (12)	0.0036 (11)
O1	0.0496 (14)	0.0502 (13)	0.0646 (16)	0.0048 (11)	0.0201 (12)	−0.0022 (11)
O2	0.0475 (13)	0.0690 (16)	0.0656 (17)	0.0010 (12)	0.0125 (12)	−0.0206 (13)
O3	0.0515 (14)	0.0615 (14)	0.0712 (17)	−0.0060 (11)	0.0322 (13)	−0.0211 (12)
O4	0.0710 (17)	0.0559 (15)	0.100 (2)	−0.0295 (12)	0.0460 (16)	−0.0189 (14)
O5	0.0614 (15)	0.0644 (15)	0.091 (2)	−0.0169 (12)	0.0435 (15)	−0.0233 (14)
O6	0.0466 (12)	0.0433 (12)	0.0700 (16)	−0.0127 (9)	0.0306 (12)	−0.0147 (11)
O7	0.089 (2)	0.101 (2)	0.139 (3)	−0.0308 (18)	0.079 (2)	−0.030 (2)
O8	0.102 (3)	0.082 (2)	0.231 (5)	−0.0489 (19)	0.101 (3)	−0.049 (3)
O9	0.141 (3)	0.0386 (14)	0.143 (3)	−0.0031 (15)	0.094 (3)	−0.0108 (16)
O10	0.116 (2)	0.0620 (16)	0.120 (3)	−0.0231 (16)	0.084 (2)	−0.0273 (16)
O11	0.0384 (13)	0.0601 (14)	0.0604 (15)	−0.0059 (10)	0.0056 (11)	0.0149 (11)
O12	0.0579 (14)	0.0512 (14)	0.0796 (18)	0.0063 (11)	0.0249 (14)	0.0197 (12)
O13	0.0321 (11)	0.0547 (13)	0.0513 (13)	−0.0040 (9)	0.0041 (9)	0.0157 (10)
O14	0.0390 (13)	0.0869 (18)	0.0759 (18)	−0.0118 (12)	−0.0185 (13)	0.0336 (15)
O15	0.0473 (14)	0.0642 (15)	0.0754 (18)	−0.0129 (12)	−0.0010 (12)	0.0166 (13)
O16	0.0299 (10)	0.0479 (11)	0.0377 (11)	−0.0010 (8)	0.0006 (8)	0.0080 (9)
O17	0.0318 (11)	0.0679 (15)	0.0616 (15)	−0.0030 (10)	0.0045 (11)	0.0026 (12)
O18	0.0487 (14)	0.0928 (19)	0.0454 (14)	0.0050 (13)	−0.0107 (11)	0.0178 (13)
O19	0.0506 (16)	0.0870 (19)	0.101 (2)	−0.0264 (13)	−0.0121 (15)	0.0424 (17)
O20	0.0700 (17)	0.0651 (15)	0.0722 (18)	−0.0028 (13)	0.0029 (14)	0.0330 (14)
O21	0.0716 (17)	0.0585 (14)	0.089 (2)	−0.0213 (12)	0.0331 (15)	−0.0060 (14)

### Geometric parameters (Å, °)

**Table:**

C1—O1	1.199 (4)	C13—C14	1.389 (4)
C1—O2	1.314 (4)	C13—C18	1.410 (4)
C1—C2	1.517 (4)	C14—C15	1.387 (4)
C2—O3	1.409 (4)	C14—H14	0.9300
C2—H2A	0.9700	C15—O16	1.338 (3)
C2—H2B	0.9700	C15—C16	1.412 (4)
C3—O3	1.355 (3)	C16—C17	1.376 (4)
C3—C4	1.380 (4)	C16—N4	1.441 (4)
C3—C8	1.408 (4)	C17—C18	1.377 (4)
C4—C5	1.392 (4)	C17—H17	0.9300
C4—H4	0.9300	C18—N3	1.450 (4)
C5—O6	1.337 (3)	C19—O16	1.414 (3)
C5—C6	1.398 (4)	C19—C20	1.496 (4)
C6—C7	1.379 (4)	C19—H19A	0.9700
C6—N2	1.455 (4)	C19—H19B	0.9700
C7—C8	1.369 (4)	C20—O15	1.192 (4)
C7—H7	0.9300	C20—O14	1.324 (4)
C8—N1	1.465 (4)	N1—O8	1.192 (4)
C9—O6	1.425 (3)	N1—O7	1.194 (4)
C9—C10	1.497 (4)	N2—O9	1.205 (3)
C9—H9A	0.9700	N2—O10	1.213 (4)
C9—H9B	0.9700	N3—O17	1.214 (3)
C10—O5	1.190 (4)	N3—O18	1.236 (3)
C10—O4	1.310 (4)	N4—O20	1.216 (3)
C11—O11	1.217 (4)	N4—O19	1.218 (4)
C11—O12	1.311 (4)	O2—H2	0.8200
C11—C12	1.512 (4)	O4—H5	0.8200
C12—O13	1.433 (3)	O12—H12	0.8200
C12—H12A	0.9700	O14—H15	0.8200
C12—H12B	0.9700	O21—H21	0.8500
C13—O13	1.343 (3)	O21—H22	0.8501
			
O1—C1—O2	125.4 (3)	C14—C13—C18	117.6 (2)
O1—C1—C2	125.2 (3)	C15—C14—C13	122.6 (3)
O2—C1—C2	109.4 (3)	C15—C14—H14	118.7
O3—C2—C1	105.3 (3)	C13—C14—H14	118.7
O3—C2—H2A	110.7	O16—C15—C14	123.7 (2)
C1—C2—H2A	110.7	O16—C15—C16	117.8 (2)
O3—C2—H2B	110.7	C14—C15—C16	118.5 (2)
C1—C2—H2B	110.7	C17—C16—C15	119.4 (2)
H2A—C2—H2B	108.8	C17—C16—N4	117.1 (3)
O3—C3—C4	123.6 (3)	C15—C16—N4	123.4 (2)
O3—C3—C8	117.5 (3)	C16—C17—C18	121.6 (3)
C4—C3—C8	118.9 (3)	C16—C17—H17	119.2
C3—C4—C5	121.6 (3)	C18—C17—H17	119.2
C3—C4—H4	119.2	C17—C18—C13	120.3 (3)
C5—C4—H4	119.2	C17—C18—N3	117.2 (3)
O6—C5—C4	124.4 (3)	C13—C18—N3	122.5 (3)
O6—C5—C6	117.8 (2)	O16—C19—C20	108.1 (2)
C4—C5—C6	117.9 (2)	O16—C19—H19A	110.1
C7—C6—C5	121.2 (3)	C20—C19—H19A	110.1
C7—C6—N2	116.4 (3)	O16—C19—H19B	110.1
C5—C6—N2	122.4 (2)	C20—C19—H19B	110.1
C8—C7—C6	120.2 (3)	H19A—C19—H19B	108.4
C8—C7—H7	119.9	O15—C20—O14	124.6 (3)
C6—C7—H7	119.9	O15—C20—C19	126.9 (3)
C7—C8—C3	120.2 (3)	O14—C20—C19	108.5 (3)
C7—C8—N1	116.7 (3)	O8—N1—O7	121.1 (3)
C3—C8—N1	123.1 (3)	O8—N1—C8	118.8 (3)
O6—C9—C10	107.2 (2)	O7—N1—C8	120.1 (3)
O6—C9—H9A	110.3	O9—N2—O10	122.2 (3)
C10—C9—H9A	110.3	O9—N2—C6	118.3 (3)
O6—C9—H9B	110.3	O10—N2—C6	119.6 (3)
C10—C9—H9B	110.3	O17—N3—O18	122.0 (3)
H9A—C9—H9B	108.5	O17—N3—C18	120.9 (3)
O5—C10—O4	124.1 (3)	O18—N3—C18	117.1 (3)
O5—C10—C9	125.0 (3)	O20—N4—O19	121.0 (3)
O4—C10—C9	110.9 (3)	O20—N4—C16	118.3 (3)
O11—C11—O12	124.4 (3)	O19—N4—C16	120.7 (3)
O11—C11—C12	124.3 (3)	C1—O2—H2	109.5
O12—C11—C12	111.2 (3)	C3—O3—C2	119.4 (2)
O13—C12—C11	106.7 (2)	C10—O4—H5	109.5
O13—C12—H12A	110.4	C5—O6—C9	119.2 (2)
C11—C12—H12A	110.4	C11—O12—H12	109.5
O13—C12—H12B	110.4	C13—O13—C12	119.2 (2)
C11—C12—H12B	110.4	C20—O14—H15	109.5
H12A—C12—H12B	108.6	C15—O16—C19	118.2 (2)
O13—C13—C14	124.3 (3)	H21—O21—H22	109.5
O13—C13—C18	118.0 (2)		
			
O1—C1—C2—O3	2.2 (5)	O13—C13—C18—C17	179.3 (2)
O2—C1—C2—O3	−179.9 (3)	C14—C13—C18—C17	−0.6 (4)
O3—C3—C4—C5	−179.4 (3)	O13—C13—C18—N3	1.2 (4)
C8—C3—C4—C5	0.7 (5)	C14—C13—C18—N3	−178.7 (2)
C3—C4—C5—O6	−178.2 (3)	O16—C19—C20—O15	−0.2 (5)
C3—C4—C5—C6	1.9 (5)	O16—C19—C20—O14	178.2 (3)
O6—C5—C6—C7	177.5 (3)	C7—C8—N1—O8	−2.7 (5)
C4—C5—C6—C7	−2.6 (5)	C3—C8—N1—O8	177.0 (4)
O6—C5—C6—N2	−3.5 (5)	C7—C8—N1—O7	177.4 (4)
C4—C5—C6—N2	176.4 (3)	C3—C8—N1—O7	−2.9 (5)
C5—C6—C7—C8	0.7 (5)	C7—C6—N2—O9	25.6 (5)
N2—C6—C7—C8	−178.4 (3)	C5—C6—N2—O9	−153.5 (4)
C6—C7—C8—C3	2.0 (5)	C7—C6—N2—O10	−153.2 (3)
C6—C7—C8—N1	−178.3 (3)	C5—C6—N2—O10	27.7 (5)
O3—C3—C8—C7	177.5 (3)	C17—C18—N3—O17	165.4 (3)
C4—C3—C8—C7	−2.7 (5)	C13—C18—N3—O17	−16.5 (4)
O3—C3—C8—N1	−2.2 (5)	C17—C18—N3—O18	−16.1 (4)
C4—C3—C8—N1	177.6 (3)	C13—C18—N3—O18	162.1 (3)
O6—C9—C10—O5	0.0 (5)	C17—C16—N4—O20	−7.4 (4)
O6—C9—C10—O4	179.4 (3)	C15—C16—N4—O20	175.1 (3)
O11—C11—C12—O13	12.5 (4)	C17—C16—N4—O19	171.4 (3)
O12—C11—C12—O13	−169.4 (3)	C15—C16—N4—O19	−6.1 (4)
O13—C13—C14—C15	179.7 (3)	C4—C3—O3—C2	6.2 (5)
C18—C13—C14—C15	−0.4 (4)	C8—C3—O3—C2	−174.0 (3)
C13—C14—C15—O16	−178.2 (2)	C1—C2—O3—C3	175.8 (3)
C13—C14—C15—C16	1.5 (4)	C4—C5—O6—C9	−2.1 (5)
O16—C15—C16—C17	178.0 (2)	C6—C5—O6—C9	177.8 (3)
C14—C15—C16—C17	−1.7 (4)	C10—C9—O6—C5	179.9 (3)
O16—C15—C16—N4	−4.5 (4)	C14—C13—O13—C12	8.7 (4)
C14—C15—C16—N4	175.7 (2)	C18—C13—O13—C12	−171.2 (3)
C15—C16—C17—C18	0.8 (4)	C11—C12—O13—C13	−173.3 (2)
N4—C16—C17—C18	−176.9 (2)	C14—C15—O16—C19	−1.6 (4)
C16—C17—C18—C13	0.4 (4)	C16—C15—O16—C19	178.7 (2)
C16—C17—C18—N3	178.6 (2)	C20—C19—O16—C15	178.0 (2)

### Hydrogen-bond geometry (Å, °)

**Table:**

*D*—H···*A*	*D*—H	H···*A*	*D*···*A*	*D*—H···*A*
O2—H2···O11^i^	0.82	1.89	2.698 (3)	168
O4—H5···O21^ii^	0.82	1.74	2.562 (3)	174
O12—H12···O1^iii^	0.82	1.92	2.730 (3)	168
O14—H15···O18^iv^	0.82	2.20	2.921 (3)	147
O14—H15···O17^iv^	0.82	2.37	3.055 (3)	141
O21—H21···O15	0.85	2.19	2.947 (4)	148
O21—H21···O10	0.85	2.66	3.084 (4)	113
O21—H22···O5	0.85	1.97	2.798 (4)	165

Symmetry codes: (i) −*x*+5/2, *y*−1/2, −*z*+1/2; (ii) −*x*, −*y*, −*z*+1; (iii) −*x*+5/2, *y*+1/2, −*z*+1/2; (iv) *x*−3/2, −*y*+1/2, *z*−1/2.
